# Studies on the Effect of Piperine on Hepatocyte Nuclear Factor 1 Alpha (HNF-1α) and Sterol Regulatory Element-Binding Protein 1c (SREBP-1c) Levels in High-Fat-Diet and Sucrose-Induced Type 2 Diabetes Mellitus Rats

**DOI:** 10.7759/cureus.54061

**Published:** 2024-02-12

**Authors:** Ummu Zuvairiya, Selvaraj Jayaraman, Kavitha Sankaran, Vishnu Priya Veeraraghavan, Gayathri R

**Affiliations:** 1 Centre of Molecular Medicine and Diagnostics (COMManD), Department of Biochemistry, Saveetha Dental College and Hospitals, Saveetha Institute of Medical and Technical Sciences, Saveetha University, Chennai, IND

**Keywords:** high-fat diet, male wistar rat, insulin resistance, gene expression, black pepper

## Abstract

Background: Piperine, a naturally occurring compound in black pepper (*Piper nigrum*), is known for its potential health benefits, including its reported enhancement of insulin sensitivity. However, the precise impact of piperine on hepatocyte nuclear factor 1 alpha (HNF-1α) and sterol regulatory element-binding protein 1c (SREBP-1c), transcription factors for insulin signaling and glucose metabolism in hepatocytes, remains unclear.

Objective: This study aims to investigate the effect of piperine, compared to metformin, on blood glucose and insulin levels by modifying the expression of hepatic HNF-1α and SREBP-1c in high-fat-diet (HFD) and sucrose-induced type 2 diabetes mellitus (T2DM) rats and in human Chang liver cells.

Methods: Adult male albino rats were categorized into four groups: group 1 as the control, group 2 as T2DM, group 3 as T2DM rats treated with piperine (40 mg), and group 4 as T2DM rats treated with metformin (50 mg). Fasting blood glucose (FBG) and serum insulin levels were measured using enzyme-linked immunosorbent assay (ELISA), while real-time polymerase chain reaction (RT-PCR) analysis was conducted to assess the mRNA expression of HNF-1α and SREBP-1c. Further, piperine was treated with normal and high glucose-induced Chang liver cells, and gene expression was analysed. Data analysis was performed using one-way analysis of variance (ANOVA), with a significance set at p<0.05.

Results: Treatment with piperine led to a notable decrease in blood glucose levels and circulating insulin when compared with T2DM rats (group 2). Additionally, piperine administration resulted in the upregulation of HNF-1α mRNA expression and downregulation of SREBP-1c mRNA levels whose effects were found to be near that of the control and standard drug metformin's effects. In vitro study also confirmed that piperine improved the HNF-1α expression and reduced the expression of SREBP-1c in Chang liver cells.

Conclusion: Our findings suggest that piperine treatment effectively regulates hyperglycemic and hyperinsulinemic insulin resistance in the liver by modulating the expression of HNF-1α and SREBP-1c. Consequently, piperine emerges as a promising candidate for therapeutic intervention in managing T2DM.

## Introduction

Diabetes mellitus (DM) is a prevalent endocrine disorder in which the body's glucose regulation is impaired. Its various types include type 1 diabetes, type 2 diabetes, maturity-onset diabetes of the young, neonatal diabetes, etc. [1]. Type 1 and type 2 diabetes mellitus (T1DM and T2DM) are the principal subcategories characterized by distinct pathophysiological mechanisms, clinical presentations, and treatment strategies [2-4]. According to projections, the global prevalence of diabetes among adults is anticipated to rise from 8.8% (424.9 million individuals) to 9.9% (628.6 million individuals) by 2045 [5]. This surge in T2DM can be linked to various factors, including aging, populations, oral contraceptives, thyroxine, adrenaline, growth hormone, the rapid expansion of urban areas, and lifestyle modifications such as high-calorie diets and sedentary habits. These trends have contributed to a widespread escalation in obesity and diabetes, making them among the most critical contemporary health challenges worldwide. Elevated fasting blood glucose (FBG) levels can be an early indicator of diabetes or prediabetes. Predicting diabetes risk based on FBG levels and other relevant factors, including body weight, serum parameters (oxidative stress, inflammatory cytokines, antioxidant enzymes), biochemical markers (lipid profile, kidney function test, liver function test), and tissue-level biomarkers (IR, IRS, PI3K, AKT, GLUT), can help individual's proactive measures to prevent or manage the condition. Despite notable progress in comprehending and managing diabetes, the disease and its associated complications continue to grow persistently [6].

Due to the relentless advancement of the disease, there is an urgent requirement to explore and scrutinize native natural resources to investigate their potential for recently identified targets and transform them into novel therapeutic interventions. The existing drug treatments for managing diabetes such as insulin secretagogues (sulfonylureas), insulin sensitizers (biguanides and thiazolidinediones), alpha-glucosidase inhibitors (miglitol, acarbose), and glucagon-like peptide-1 (GLP-1) agonists possess certain constraints, underscoring the necessity for safer and more potent antidiabetic medications. The confidence in the safety of natural remedies in contrast to synthetic drugs has gained considerable traction recently, contributing to a noteworthy surge in the utilization of phytopharmaceuticals. Piperine, an alkaloid sourced from *Piper nigrum* commonly known as black pepper, is a flowering vine belonging to the family Piperaceae. It is cultivated for its fruit, which is dried and used as a spice and seasoning worldwide. *Piper nigrum* is recognized as one of the initial naturally derived compounds acknowledged for its bioenhancing attributes [7,8]. Its diverse array of pharmacological potentials, akin to curcumin, is associated with a challenge concerning its restricted bioavailability (30-200%). The evaluation of piperine's therapeutic effects has also been considered in the context of its clinical approval as an antidiabetic agent [9]. It aids in amplifying drug bioavailability by obstructing the biotransformation processes in the liver and intestines [10]. One of the earliest investigations into piperine, to the best of current knowledge, delves into the combined use of bioactive compounds of *Piper nigrum* [10]. Bioactive compounds of *Piper nigrum* L have been proven to have various health benefits, including the mitigation of insulin resistance, enhancement of hepatic steatosis anticancer activity, and increase in the activity of digestive enzymes, exhibiting antimicrobial, antioxidant, and anti-inflammatory activities. Nevertheless, the mechanisms by which piperine operates against high-fat-diet (HFD) and sucrose-induced T2DM remain unexplored.

Hyperinsulinemia is a condition characterized by elevated levels of insulin in the blood. It is often associated with insulin resistance, a critical factor in the development of T2DM. Managing hyperinsulinemia and preventing its progression to T2DM can involve various strategies, including lifestyle changes and using natural compounds with potential beneficial effects. Hepatocyte nuclear factor 1 alpha (HNF-1α) is a transcription factor involved in the regulation of genes related to insulin sensitivity and glucose metabolism in the liver. Altered HNF-1α function could contribute to insulin resistance, a key feature of T2DM. HNF-1α comprises diverse homeodomains that can bind to DNA as either individual or paired units, thereby regulating the transcription of numerous genes [11]. Sterol regulatory element-binding protein (SREBP), recognized as a master regulator of lipid homeostasis, oversees the expression of a wide range of enzymes essential for intracellular cholesterol synthesis. Moreover, the SREBP-1c isoform is primarily subject to transcriptional regulation by insulin [7]. SREBP-1c is a transcription factor that plays a significant role in the regulation of lipid metabolism, including the synthesis of fatty acids and triglycerides. While its primary function is related to lipid metabolism, SREBP-1c also has indirect effects on glucose homeostasis and insulin resistance through its influence on lipid metabolism and other signaling pathways. It promotes the expression of genes involved in fatty acid synthesis, such as fatty acid synthase (FASN). This leads to an increased production of fatty acids, which can have several implications for glucose homeostasis and insulin resistance. Hence, our study explores the capacity of piperine to manage hyperglycemia by modulating HNF-1α and SREBP-1c expression in an HFD and sucrose-induced insulin resistance/T2DM using an in vivo experimental animal model and in vitro cell line (Chang liver cells) model.

## Materials and methods

Animals

Male albino Wistar rats, aged 150 days and weighing 180 and 200 grams, were housed under controlled conditions at the Biomedical Research Unit and Laboratory Animal Centre (BRULAC), Saveetha Dental College (SDC), Saveetha Institute of Medical and Technical Sciences (SIMATS), Tamil Nadu, India (protocol number: BRULAC/SDCH/SIMATS/IAEC/07-2019/028). The animals were divided into four groups (n=6): group 1 served as the control and received corn oil injections (1 ml) intraperitoneally daily, group 2 was subjected to an HFD along with sucrose, and group 3 received piperine (40 mg), while group 4 was treated with metformin (50 mg). Following the treatment, the animals were anesthetized using ether, and blood samples were collected and sera separated, which were then stored at -80°C for further analysis. Moreover, liver tissue was extracted from the experimental groups to evaluate various parameters.

T2DM development in animals

Diabetes was induced by HFD and sucrose as per the standard methods.

FBG

Following an overnight fasting period, firstly, blood samples were collected from the rat's tail tip, and the FBG levels were evaluated using CareSens N blood glucose test strips (iSENS Biosensors India Private Limited, Gurgaon, India), with results presented in mg/dL. 

Serum insulin

A commercially accessible rat insulin enzyme-linked immunosorbent assay (ELISA) kit (Krishgen Biosystems, Mumbai, India) was employed for the quantification of serum insulin levels. The detection range of the kit was 0.1-64 ng/mL. The insulin concentration was expressed in µIU/mL.

mRNA expression analysis of HNF-1α and SREBP-1c, relative to β-actin genes by real-time polymerase chain reaction (RT-PCR)

We utilized the RNAiso Plus reagent (Takara Bio, Kusatsu, Shiga, Japan) for RNA extraction from the liver tissue of both control and diabetic rats. The 100 mg RNA tissue was homogenized, followed by centrifugation and resuspension in a 70% ethanol solution. The RNA pellet was then dissolved in nucleus-free water and quantified at nanodrop. About 2 µg of RNA was taken and reverse-transcribed into cDNA using the Reverse Transcriptase Core kit (Eurogentec, Seraing, Belgium). The primers utilized in this study are listed in Table 1. The SYBR Green Master Mix (Takara Bio, Kusatsu, Shiga, Japan) and housekeeping gene (β-actin) were used in an RT-PCR system (Bio-Rad C1000 Touch Thermal Cycler, Bio-Rad Laboratories Ltd. (Bio-Rad House, Herts, UK)) to amplify the interest genes under the following reaction conditions: initial denaturation at 95°C for five minutes, followed by 40 cycles of denaturation at 95°C for 30 s, annealing at 60°C for one minute, and extension at 72°C for 30 s each. The analyses of melt and amplification curves were carried out to calculate the relative quantification.

**Table 1:** Primer sequence details SREBP-1c: sterol regulatory element-binding protein 1c; HNF-1α: hepatocyte nuclear factor 1 alpha

S. no.	Gene name	Sequence
1	SREBP-1c	FW-5’-GACGACGGAGCCATGGATT-3’, RW-5’-GGGAAGTCACTGTCTTGGTTGTT-3’
2	HNF-1α	FW-5’-GACGACGGAGCCATGGATT-3’, RW-5’-GGGAAGTCACTGTCTTGGTTGTT-3’
3	β-Actin	FW-5’-AAGTCCCTCACCCTCCCAAAAG-3’, RW-5’-AAGCAATGCTGTCACCTTCCC-3’

In vitro study on Chang liver cells

Cell Culture

The Chang liver cell line was obtained from the National Centre for Cell Science (NCCS) located in Pune, India.

High-Glucose Treatment and Cell Viability

Chang liver cells underwent cultivation in Dulbecco's Modified Eagle Medium (DMEM) supplemented with 10% fetal bovine serum (FBS), glutamine, 4-(2-hydroxyethyl)-1-piperazineethanesulfonic acid (HEPES), and streptomycin/penicillin (100 mg/mL, 100 units/mL). This culture took place within a 5% CO_2_ incubator at 37°C. The Chang liver cell treatment involved the addition of either 5.5 mM glucose (normal glucose (NG)) or 33 mM glucose (high glucose (HG)), with piperine (40-120 μM). Briefly, after 24-hour exposure to piperine, the culture plates were aspirated, and the cells were washed with phosphate-buffered saline. Subsequently, 200 μl of DMEM containing 5 mg/mL 3-(4,5-dimethylthiazol-2-yl)-2,5-diphenyltetrazolium bromide (MTT) was added to each well and incubated for four hours at 37°C. Following incubation, the medium with MTT was removed, and 200 μl of dimethyl sulfoxide (DMSO) was added and agitated for 10 minutes. Subsequently, 100 μl from each well was transferred to an ELISA reader plate, and the absorbance of the converted dye was measured at 570 nm.

mRNA Expression by RT-PCR

The total RNA extraction from both NG and HG, HG+piperine, and HG+metformin was accomplished using the Total RNA Isolation Reagent (TRIR) kit (Thermo Scientific, Cambridge, UK) sourced from Ab gene home in the United Kingdom. The quantification of the extracted RNA was conducted using spectrophotometry, and the results were expressed in micrograms (μg). Following this, cDNA synthesis was performed using 2 μg of total RNA with the Reverse Transcriptase Core kit from Eurogentec (Seraing, Belgium), following the manufacturer's instructions. Real-time PCR was carried out using the SYBR Green Master Mix from Takara Bio in combination with specific forward and reverse primers for the target genes. The obtained results were graphically represented using the CFX96 Touch Real-Time PCR Detection System (Bio-Rad Laboratories, Hercules, CA) from the United States. Relative quantification was achieved through the analysis of melt and amplification curves.

Statistical Analysis

The results were presented as the mean±standard error of the mean (SEM) from three distinct experiments conducted in triplicate. Statistical analysis involved the utilization of one-way analysis of variance (ANOVA), with p-values less than 0.05 being considered indicative of statistically significant findings.

## Results

Effect of piperine on FBG in HFD and sucrose-induced T2DM rats

**Figure 1:**
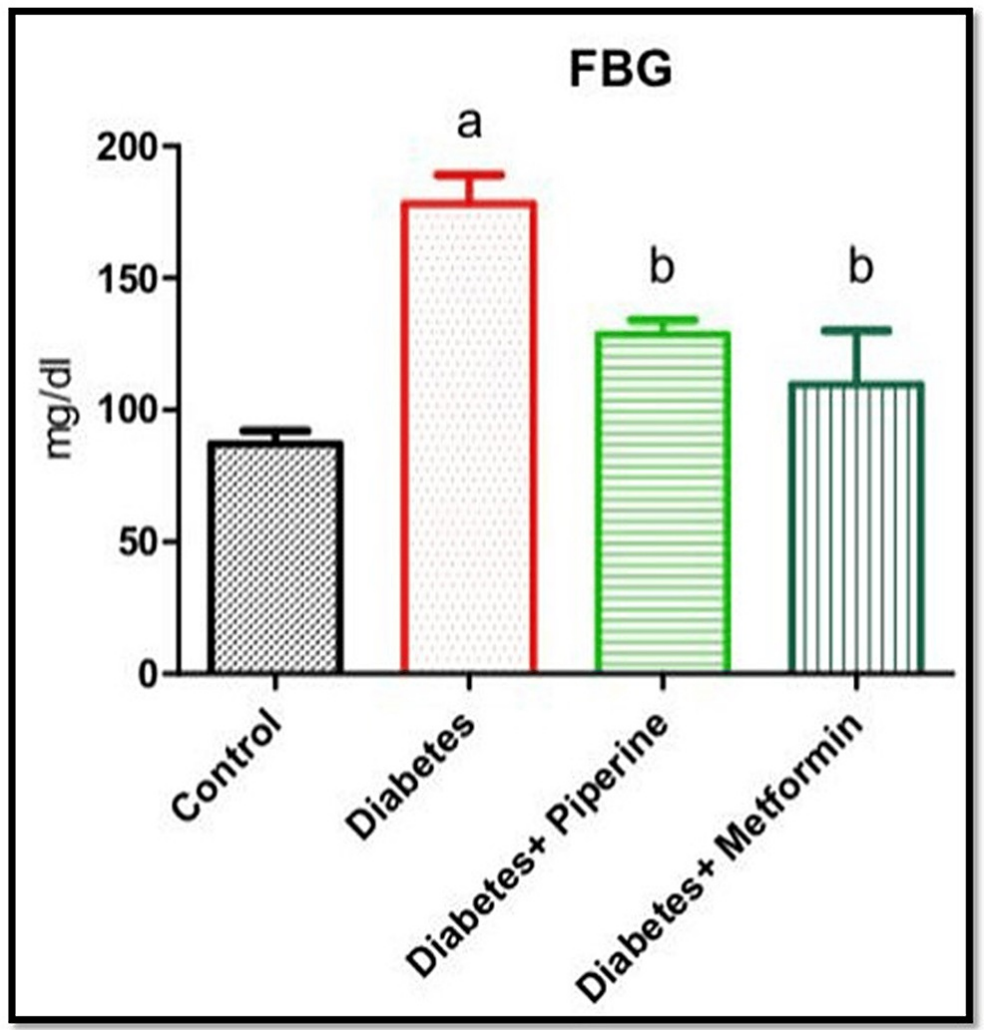
FBG Every bar illustrates the mean±SEM derived from six animals. a denotes significance with the control group; b signifies a comparison with rats in the diabetes group FBG: fasting blood glucose; SEM: standard error of the mean

FBG levels are a crucial parameter for diagnosing and managing diabetes [12]. Hence, in the present study, we measured FBG after overnight fasting in order to check whether piperine can reduce hyperglycemia [12]. As depicted in Figure 1, HFD caused a significant rise (p<0.05) in the FBG when compared to the control. The concentration of FBG was found to be 90, 192, 140, and 115 mg/dL in groups 1, 2, 3, and 4, respectively. However, piperine treatment brought down the FBG levels to the normal range, whose effect was found to be equal to that of the standard drug metformin level.

Effect of piperine on fasting serum insulin concentration in HFD and sucrose-induced T2DM rats

**Figure 2:**
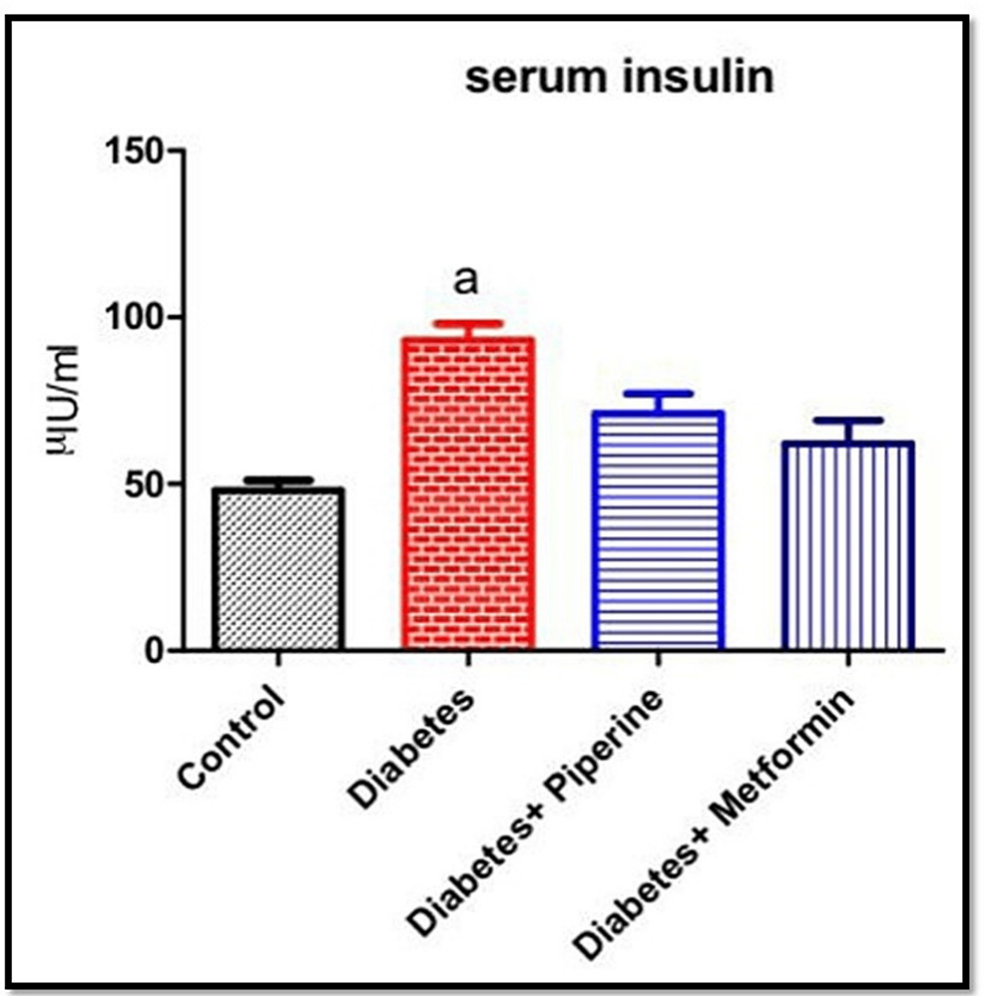
Serum insulin level Every bar illustrates the mean±SEM derived from six animals. a denotes significance with the control group SEM: standard error of the mean

The fasting insulin test measures the level of insulin in the blood after a period of fasting and can indicate a trend toward diabetes. In this study, HFD and sucrose induction significantly induced (p<0.05) hyperinsulinemia, but piperine treatment reduced the same, suggesting that piperine treatment clearly indicates that it may have the ability to manage T2DM (Figure 2). The concentrations of fasting serum insulin in the groups were 49, 95, 70, and 60 µIU/mL in groups 1, 2, 3, and 4, respectively.

Effect of piperine on the mRNA expressions of HNF-1α in the liver of HFD and sucrose-induced T2DM rats

**Figure 3:**
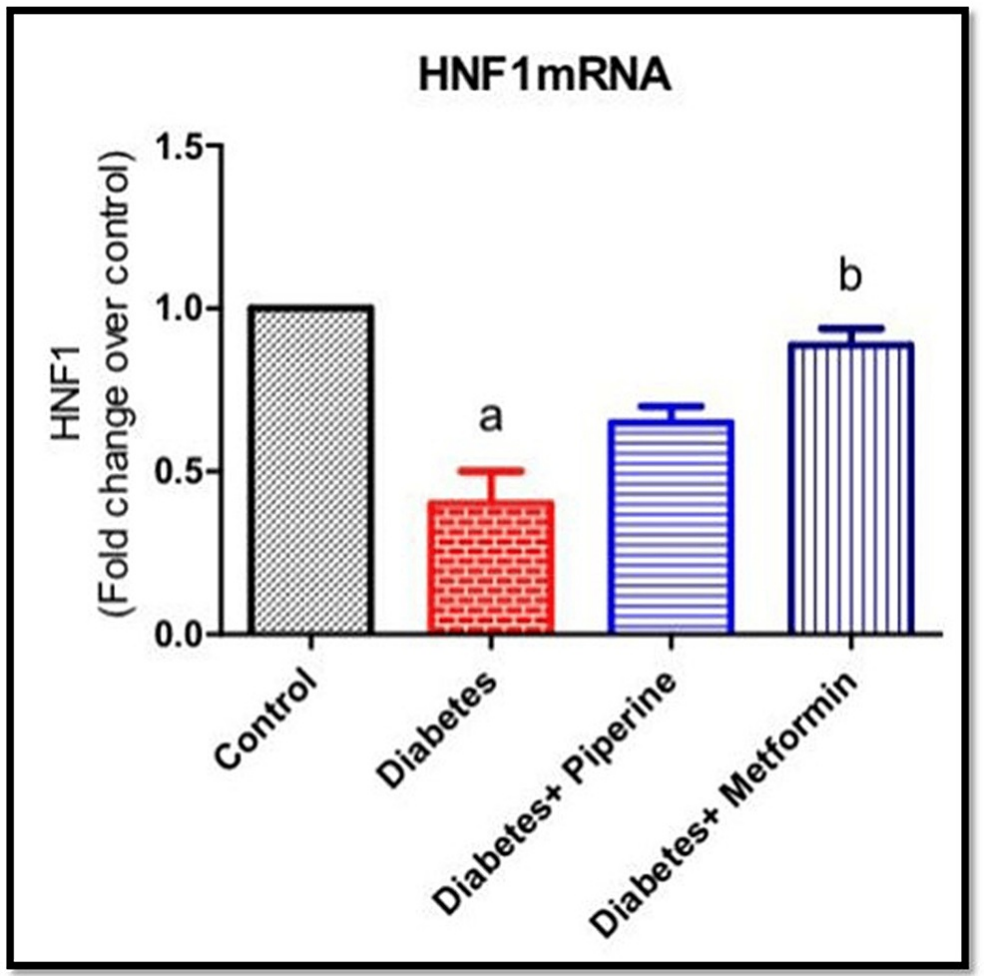
mRNA expression of HNF-1α in control, diabetic, and piperine-treated groups Every bar illustrates the mean±SEM derived from six animals. a denotes significance with the control group; b signifies a comparison with rats in the diabetic group HNF-1α: hepatocyte nuclear factor 1 alpha; SEM: standard error of the mean

HNF-1α is a transcription factor involved in the regulation of genes related to insulin sensitivity and glucose metabolism in the liver [13]. Altered HNF-1α function could contribute to insulin resistance, a key feature of T2DM [13]. In this study, we wanted to find out whether piperine can regulate glucose homeostasis via HNF-1α expression in hepatocytes. We measured the HNF-1α mRNA levels by real-time PCR method using gene-specific primers. As depicted in Figure 3, HNF-1α mRNA expression was significantly (0.8-fold) reduced in HFD-induced T2DM rats (p<0.05). Fascinatingly, an oral dose of piperine improved the expression of HNF-1α mRNA level when compared to the diabetic group. The fold change expressions over control on HNF-1α mRNA were 1-fold, 0.35-fold, 0.7-fold, and 0.95-fold in groups 1, 2, 3, and 4, respectively.

Effect of piperine on the mRNA expressions of SREBP-1c in the liver of HFD and sucrose-induced T2DM rats

**Figure 4:**
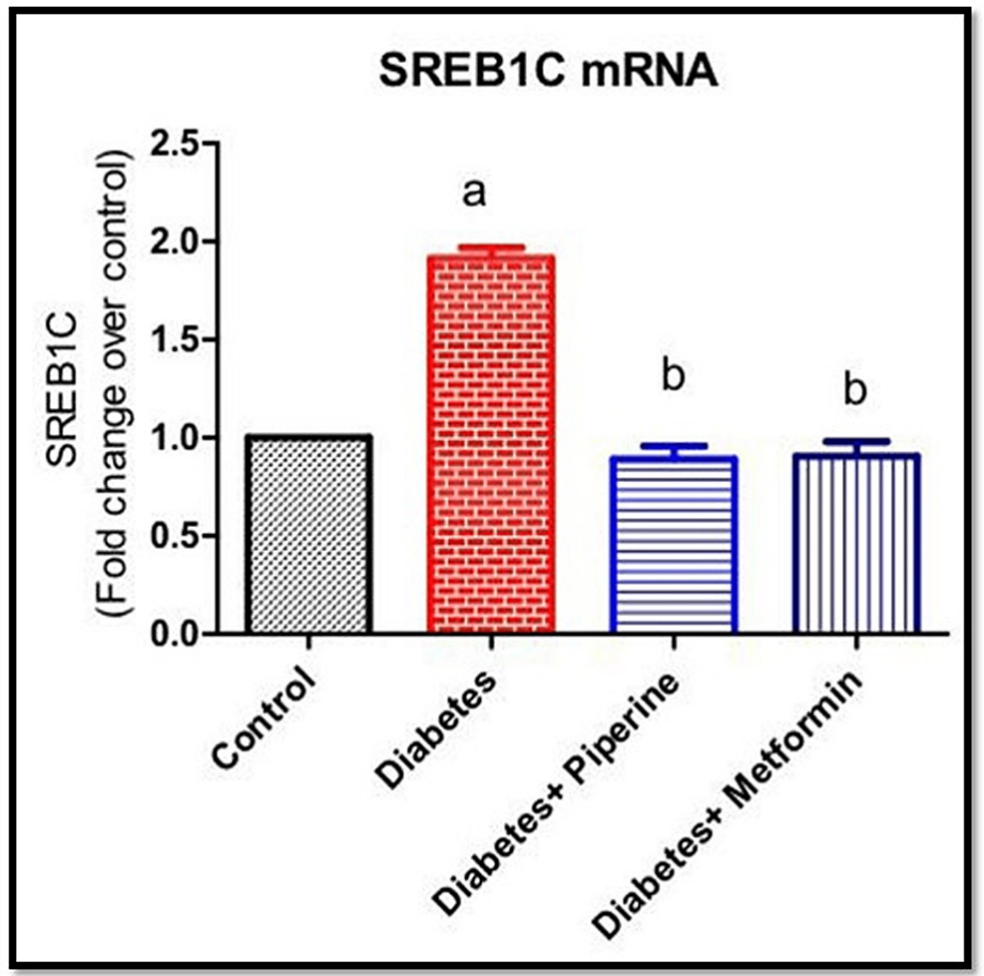
mRNA expression of SREBP-1c All the bar depicts mean±SEM of six animals. a denotes significance with the control group; b signifies a comparison with rats in the diabetic group SREBP-1c: sterol regulatory element-binding protein 1c; SEM: standard error of the mean

SREBP-1c is a transcription factor that plays a significant role in the regulation of lipid metabolism, including the synthesis of fatty acids and triglycerides [14]. While its primary function is related to lipid metabolism, SREBP-1c also has indirect effects on glucose homeostasis and insulin resistance through its influence on lipid metabolism and other signaling pathways. It promotes the expression of genes involved in fatty acid synthesis, such as FASN. This leads to an increased production of fatty acids, which can have several implications for glucose homeostasis and insulin resistance [14]. In this study, in order to find out the involvement of SREBP-1c in insulin resistance, we measured SREBP-1c mRNA levels in both control and treated animals. This finding implies that HFD-induced rats showed a significant elevation (p<0.05) in the mRNA level, whereas piperine treatment was able to reduce the mRNA expression of SREBP-1c in T2DM rats (Figure 4). The fold change expressions of SREBP-1c in all the groups were 1-fold, 1.95-fold, 0.82-fold, and 0.91-fold in groups 1, 2, 3, and 4, respectively.

Effect of piperine on cell viability in untreated and HG-treated human Chang liver cells

**Figure 5:**
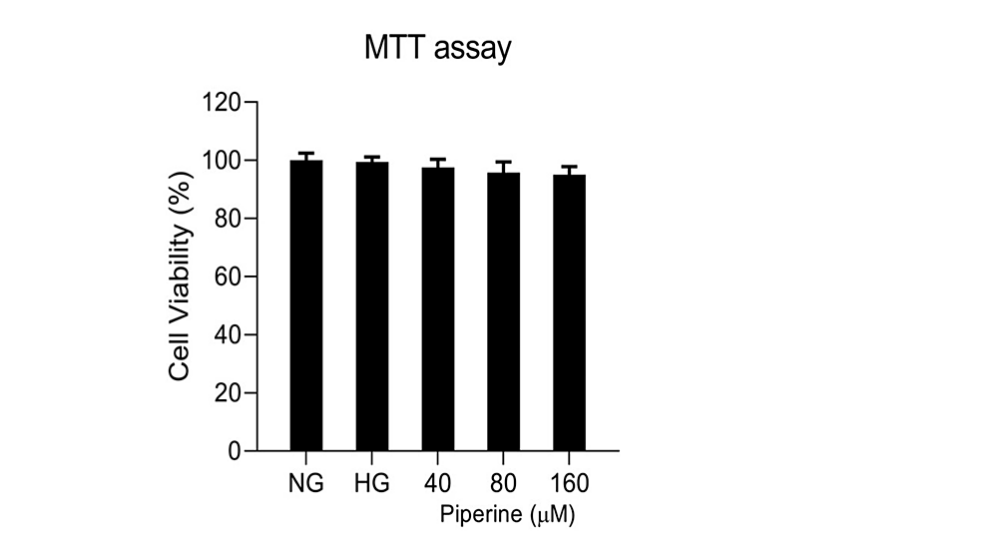
Effect of piperine on cell viability in Chang liver cell lines. The cells were treated with different doses of piperine (40, 80, and 160 μM) in HG-treated cells HG: high glucose; MTT: 3-(4,5-dimethylthiazol-2-yl)-2,5-diphenyltetrazolium bromide

Figure 5 represents the dose-dependent effects of piperine on the cell viability of the human liver cell line (Chang liver cells). The cells were treated with three different doses of piperine (40, 80, and 160 μM) in control (NG) and HG-treated cells. No significant change was observed in the viable liver cells in all groups studied, and the percentages of viable cells were found to be 100%, 99.9%, 97.4%, 95.75%, and 95.05% in the control (NG) and HG-, HG+40 μM piperine-, HG+80 μM piperine-, HG+160 μM piperine-treated groups (Figure 5).

Effect of piperine on HNF-1α and SREBP-1c gene expression in human Chang liver cells by real-time PCR

**Figure 6:**
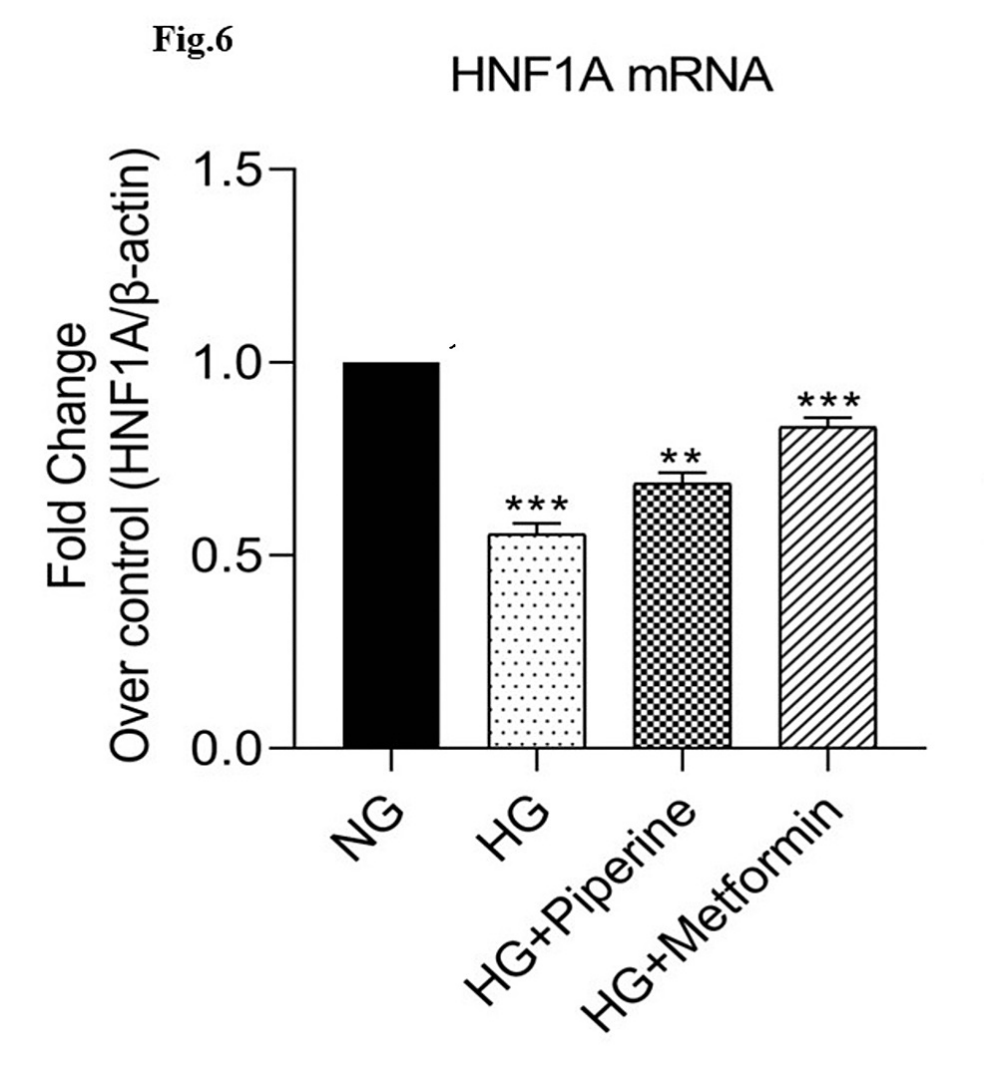
Effect of piperine on HNF-1α-treated human Chang liver cells. mRNA expression was done by real-time PCR analysis and expressed as fold change over control HNF-1α: hepatocyte nuclear factor 1 alpha; PCR: polymerase chain reaction

**Figure 7:**
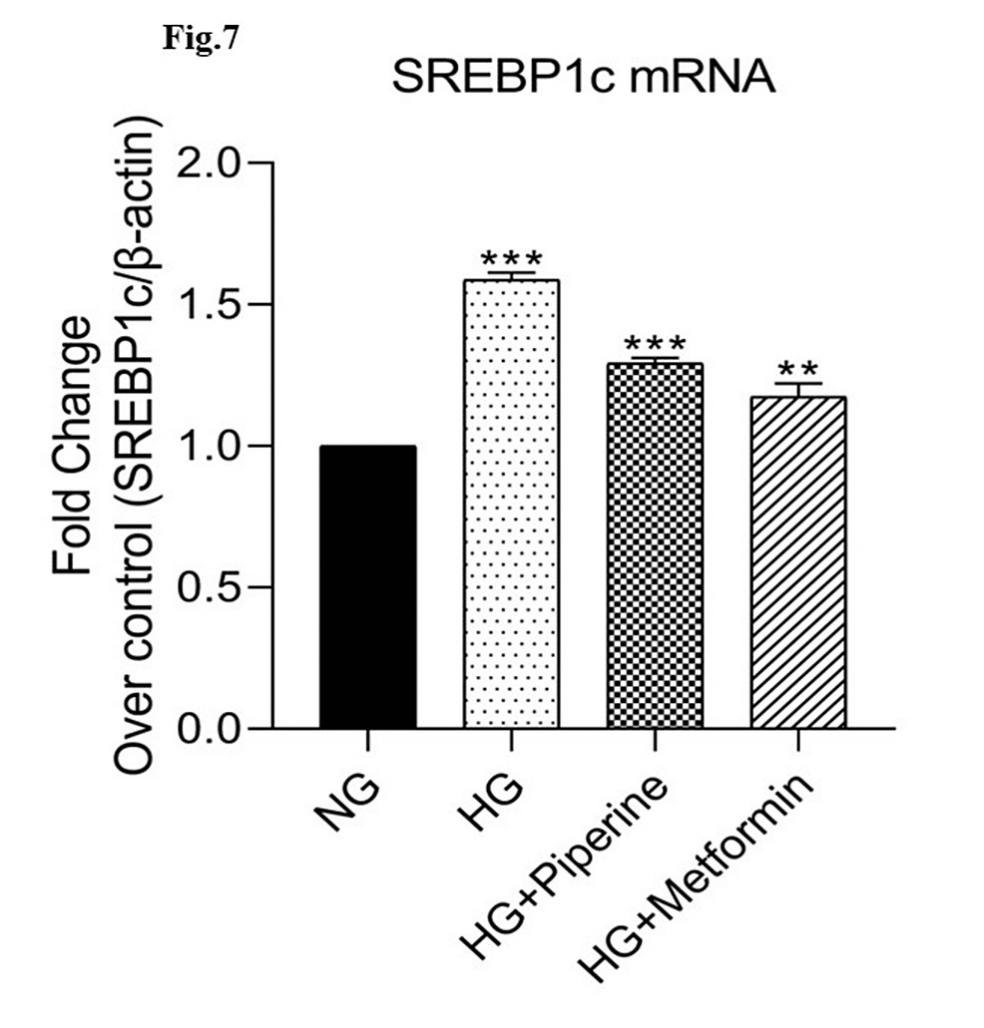
Effect of piperine on SREBP-1c-treated human Chang liver cells. mRNA expression was done by real-time PCR analysis and expressed as fold change over control SREBP-1c: sterol regulatory element-binding protein 1c; PCR: polymerase chain reaction

Figure 6 and Figure 7 show the mRNA expression of HNF-1α and SREBP-1c in response to different doses of piperine. Normal Chang liver cells were treated with 160 μM concentration of piperine. After the cells were harvested and 1 mL RNA isolation reagent was added proceeded with RNA isolation and cDNA conversion, mRNA expressions of HNF-1α and SREBP-1c were analysed by real-time PCR. In HG-treated cells, HNF-1α mRNA expression was 0.55-fold decreased compared to NG-treated cells, whereas in the cells treated with 160 μM piperine and metformin, HNF-1α expression significantly (p<0.05) improved 0.68-fold and 0.85-fold (Figure 6). SREBP-1c expression was 1.56-fold increased in HG-treated cells compared to control. Piperine treatment significantly reduced the expression to 1.31-fold and 1.12-fold in piperine- and metformin-treated HG-induced cells (Figure 7).

## Discussion

Being a metabolic disorder, a wide range of confounding factors promote the onset of diabetes. With lifestyle modification and sedentary lifestyle as the primary risk factors for acquiring T2DM, we intended to check whether the intake of a high-calorie diet (rich in fat and sugar) could possibly result in impaired glucose metabolism. Also simultaneously, our motive was to identify a natural herbal-based compound as a therapeutic approach in combating this metabolic alteration. The active constituents derived from medicinal plants have undergone extensive research to assess their potential use as drugs for treating various diseases or as primary components in pharmacological agents [15-17]. From a therapeutic view, our investigation explored the antidiabetic effects of the natural herbal medicine piperine, an active component of *Piper nigrum*. In this accord, at the end of the study, the rats fed with HFD and sucrose water showed a peaking in circulating blood glucose levels (Figure 1). However, upon treatment with piperine, there was a restoration of glucose levels near to normal significantly when compared to the control and diabetic groups [18,19]. Impairment of the blood glucose level, both during fasting and post prandial, is a milestone symptom of the onset of T2DM. Thus, it was proved that constant intake of HFD along with food rich in sugar can impair the glucose uptake by the cells. We then assessed the serum insulin level among all the groups. The results showed that the serum insulin levels were elevated in the HFD+sucrose diabetic group than the control significantly. Hyperinsulinemia is a condition characterized by T2DM condition. Hyperinsulinemia and the development of insulin resistance in response to HFD are interconnected processes that contribute to the development of T2DM and other metabolic disorders [20]. An increase in the serum insulin levels can create resistance in the insulin receptors (IR), thereby affecting its binding to IR, thus affecting the downstream signaling and ultimately glucose uptake from circulation. Excessive consumption of dietary fats can lead to the accumulation of lipids (fats) within cells, particularly in the skeletal muscle and liver cells [20]. This intracellular lipid accumulation can interfere with insulin signaling pathways, making it more difficult for insulin to promote glucose uptake. However, this insulin-resistant condition was neutralized upon administration of piperine to the diabetic rats by lowering the serum insulin level. This shows that the HFD+sucrose diet promoted insulin resistance in the cells which on the other hand elevated the circulating free glucose level. Our next parameter assessed was the gene expression of HNF-1α and SREBP-1c. The reason for choosing these specific genes in our study is because they both play essential roles in insulin signaling and are relevant to the effects of HFD on metabolic regulation. HNF-1α is an important metabolic regulating gene that codes for a number of proteins involved in a number of physiological roles. Impairment of this gene will result in the loss of maintaining blood glucose homeostasis by controlling the production and release of glucose into the bloodstream [21]. Likewise, SREBP-1c is a transcription factor that plays a central role in promoting the liver's production of fatty acids and triglycerides when dietary carbohydrates are in excess [22]. Excessive activation of SREBP-1c in response to an HFD can lead to the accumulation of fat within hepatocytes (hepatic steatosis). This condition is associated with insulin resistance, where cells become less responsive to insulin's effects. In our study, the mRNA expressions of these two genes were abnormally altered in the HFD+sucrose-induced diabetic group significantly when compared to the control group. It has been reported that various phytocompounds such as curcumin, berberin, resveratrol, and epigallocatechin gallate (EGCG) have been shown to improve insulin sensitivity through the regulation of HNF-1α and SREBP-1c due to their anti-inflammatory and antioxidant potentials [23,24].

Further, we checked if piperine can restore HNF-1α and SREBP-1c expression; hence, we performed an RT-PCR analysis using gene-specific primers. Results of this study show that piperine effectively upregulates HNF-1α while reducing the expression of SREBP-1c in hepatocytes.

To check the efficacy of our drug of interest, piperine, an internal control (metformin) was tested simultaneously along with other groups for all the abovementioned parameters. Metformin was chosen as a positive control as it is a commercially available standard antidiabetic drug. In FBG, administering metformin to rats showed a downfall of circulating free glucose units to normal levels. In the serum insulin analysis, the metformin group again restored the serum insulin level almost near to control. Furthermore, it is noteworthy to mention that the effects of metformin on HNF-1α and SREBP-1c are currently the subject of ongoing research. Investigating the potential influence of metformin on these factors may provide additional insights into its broader impact on hepatic function and metabolic regulation. Hence, metformin was chosen as the standard drug in this study. While the impact of piperine on HNF-1α and SREBP-1c in the liver has not been extensively explored in existing literature, the observed modulation of HNF-1α and SREBP-1c expression in the current study could be attributed to the potential anti-inflammatory and antioxidant properties of piperine that may play a role in regulating insulin sensitivity within the liver.

Limitations of the study

Though the current study offers experimental evidence of the therapeutic impact of piperine on reducing glucose levels through the modulation of HNF-1α and SREBP-1c gene expression as well as FBG and circulating insulin levels in rats induced with an HFD, the evaluation of protein expression of HNF-1α and SREBP-1c in hepatocytes was not conducted. These parameters could serve as complementary findings, supporting and demonstrating the role of piperine's exact mechanisms of action in the liver.

## Conclusions

The current findings demonstrate that piperine effectively diminishes hyperglycemia and hyperinsulinemia, ameliorating insulin resistance in the hepatocytes of HFD and sucrose-induced T2DM rats and human Chang liver cells. This effect is achieved by regulating key transcription factors such as HNF-1α and SREBP-1c, with outcomes comparable to those of the standard drug metformin. These results suggest that piperine is beneficial in managing DM, indicating its potential as an effective therapeutic agent. 
